# Neural effects of deep brain stimulation on reward and loss anticipation and food viewing in anorexia nervosa: a pilot study

**DOI:** 10.1186/s40337-023-00863-3

**Published:** 2023-08-21

**Authors:** M. S. Oudijn, J. T. W. Linders, A. Lok, P. R. Schuurman, P. van den Munckhof, A. A. van Elburg, G. A. van Wingen, R. J. T. Mocking, D. Denys

**Affiliations:** 1 Department of Psychiatry and Neurosurgery, Amsterdam University Medical Centers (AUMC)-Academic Medical Center (AMC), University of Amsterdam (UvA), PO Box 22660, 1100 DD Amsterdam, The Netherlands; 2 Faculty of Social Sciences, University of Utrecht, Utrecht, The Netherlands

**Keywords:** Anorexia nervosa, Deep brain stimulation, Treatment-refractory, Body Mass Index, Neurosurgery, Clinical trial, Functional neuroimaging, Brain networks, Reward

## Abstract

**Background:**

Anorexia nervosa (AN) is a severe and life-threatening psychiatric disorder. Initial studies on deep brain stimulation (DBS) in severe, treatment-refractory AN have shown clinical effects. However, the working mechanisms of DBS in AN remain largely unknown. Here, we used a task-based functional MRI approach to understand the pathophysiology of AN.

**Methods:**

We performed functional MRI on four AN patients that participated in a pilot study on the efficacy, safety, and functional effects of DBS targeted at the ventral limb of the capsula interna (vALIC). The patients and six gender-matched healthy controls (HC) were investigated at three different time points. We used an adapted version of the monetary incentive delay task to probe generic reward processing in patients and controls, and a food-specific task in patients only.

**Results:**

At baseline, no significant differences for *reward anticipation* were found between AN and HC. Significant group (AN and HC) by time (pre- and post-DBS) interactions were found in the right precuneus, right putamen, right ventral and medial orbitofrontal cortex (mOFC). No significant interactions were found in the food viewing task, neither between the conditions high-calorie and low-calorie food images nor between the different time points. This could possibly be due to the small sample size and the lack of a control group.

**Conclusion:**

The results showed a difference in the response of reward-related brain areas post-DBS. This supports the hypotheses that the reward circuitry is involved in the pathogenesis of AN and that DBS affects responsivity of reward-related brain areas.

*Trial registration* Registered in the Netherlands Trial Register (https://www.trialregister.nl/trial/3322): NL3322 (NTR3469).

**Supplementary Information:**

The online version contains supplementary material available at 10.1186/s40337-023-00863-3.

## Abstract (plain language summary)

Anorexia Nervosa (An) is a severe eating disorder with many, sometimes life-threatening, complications. A substantial number of AN patients do not respond to the available treatment options and remain chronically ill or even die as a consequence of the AN. Because part of the causes of AN may reside in the brain, we studied the efficacy and safety of a potential new treatment option for AN, namely deep brain stimulation (DBS). DBS has proven to be an effective treatment option for movements disorders like Parkinson’s Disease and other psychiatric disorders such as obsessive compulsive disorder. Our previous pilot study and other research have shown that DBS leads to improvements in weight, mood, anxiety, and eating disorder symptoms. In this substudy, we examined the effects of DBS on specific brain circuitries that are implicated in AN. We conducted brain scans (fMRI) to measure brain activity while patients performed tasks. We observed a difference in brain response when we compared scans taken before and after the DBS, which supports our thoughts on the involvement of specific parts of the brain in AN.

**Supplementary Information:**

The online version contains supplementary material available at 10.1186/s40337-023-00863-3.

## Introduction

Anorexia nervosa (AN) is a severe and life-threatening psychiatric disorder. Deep brain stimulation (DBS) has been proposed as a promising last-resort treatment option for severe therapy refractory AN-patients [1]. A recent meta-analysis showed overall beneficial effects of DBS on weight, eating disorder, depression and anxiety symptoms, as well as quality of life [2]. A better understanding of the working mechanisms of DBS in AN would improve our understanding of the pathophysiology AN and enhance DBS therapy by optimizing patient and target selection.

Studies on DBS in AN have explored diverse targets including the subcallosal cingulate cortex (SCC), nucleus accumbens (NAcc) and ventral anterior limb of the internal capsule (vALIC) [1, 3–6]. Interestingly, different targets have shown comparable clinical effects, suggesting that DBS is effective at different targets and normalizes wider aberrant network activity in the brain. This aligns with the concept of connectomic DBS, where different DBS targets relate to similar pathophysiologically relevant white matter tracts [7].

One important circuit that may play a role in the clinical effects of DBS in AN is the reward system which has been proposed as a key brain circuit in the pathophysiology of AN [8–17]. The reward circuit encompasses multiple brain regions including the ventral striatum, insula and prefrontal cortex. In AN, the reward system processes the *motivation* for eating, the *hedonic* experience of food, and the *value* of specific food items. Neuroimaging studies have demonstrated dysfunctional activation in structures associated with salience and reward networks related to emotional and reward processing, as well as in a cortical cognitive circuit related to selective attention and planning, in both individuals with AN and those who have recovered from the disorder [17]. These brain regions are part of the cortico-striatal-limbic neurocircuit, which is also implicated in other reward related psychiatric disorders such as obsessive–compulsive disorder (OCD) [18, 19].

Reward-based neurobiological models suggest that AN is characterized by deficient reward processing and enhanced punishment processing. AN symptoms are thought to be maintained by reward-based learning, where abnormal eating- and weight-related cognitions alter reward processing. One hypothesis is that AN patients have a diminished sense of reward towards food and a decreased motivation for food consumption, [20–22]. Additionally, studies have found an exaggerated response to losses in executive and striatal regions using a monetary guessing task [14], as well as increased activation in the insula and cingulate during loss anticipation in a monetary incentive delay task, indicating hypersensitivity to punishment in general [23]. It is hypothesized that in AN, cues compatible with the illness (such as weight-loss behaviors, thinness and excessive exercising) become positively associated with reward while healthy cues (such as seeing, tasting and smelling food and foraging behavior) lose their primary rewarding properties, and instead become aversive [9, 24, 25]. These findings are accompanied by studies that have found altered activation in areas associated with cognitive control and rigidity [26].

Despite the importance of the reward circuit in AN and impact of DBS, the effects of DBS on the reward circuit in AN remain unknown. One study demonstrated that DBS reduces maladaptive activity and connectivity of the stimulated regions in OCD patients [27]. Previous studies on DBS in AN have used positron emission tomography (PET) to investigate effects of DBS on resting glucose metabolism [4, 28, 29]. One study found significant reduced activity of the subcallosal and anterior cingulate and significant hyperactivity of parietal structures including the supramarginal gyrus and cuneus, following treatment with DBS using PET. This suggests that a focal intervention can have a broad effect on neural structures downstream, albeit slightly different, but relevant to key illness-related structures structures [4, 28]. An F-FDG PET study in patients with AN showed that the pre-DBS found hypermetabolism in frontal lobe, hippocampus, lentiform nucleus, left insula and left subcallosal gyrus decreased after NAcc-DBS [29]. A study using diffusion magnetic resonance imaging (dMRI) and deterministic multi-tensor tractography in patients with AN undergoing DBS identified widely-distributed differences in subcallosal white matter (SCC) connectivity, consistent with heterogenous clinical disruptions [30].

In this study we utilized task-based functional MRI to investigate changes in activity in the cortico-striatal-limbic circuit during an adapted version of the monetary incentive delay (MID) task, employing monetary reward and loss as motivational rewarding stimuli [31]. Additionally, we employed a food viewing task, where subjects were presented with high- and low-calorie food pictures and neutral pictures. The MID task was chosen to explore non-food related changes in the reward response, while the food viewing task aimed to examine responses to disease-specific stimuli.

Our hypotheses were twofold:

1. Patients with AN would exhibit heightened activation in reward-related brain areas in monetary tasks before DBS compared to healthy controls, particularly with regard to losses (indicative of the heightened sensitivity to punishment). We expected this heightened activation to normalize following DBS.
2. We anticipated increased activation in reward-related areas during the food viewing tasks before DBS, especially in response to high-calorie food pictures compared to low-calorie or neutral pictures. This might indicate a heightened response to aversive cues (high-calorie food pictures) in AN. Additionally, we expected low-calorie pictures to elicit higher activation in the cortico-striatal circuit, as these cues are thought to be rewarding in AN patients. Furthermore, we hypothesized a possible hyperactivation of areas associated with cognitive control in AN before DBS [17, 32]. We speculated that aberrant reward-response to high-calorie and low-calorie food pictures would normalize after DBS.

## Methods

### Study design

We conducted this study at the Department of Psychiatry and the Department of Neurosurgery of the Amsterdam UMC (Amsterdam University Medical Centers), location AMC [1]. The Medical Ethical Committee (MEC) of the Amsterdam UMC, location AMC, approved the study (MEC number: 2012_169). We used an open label intervention clinical trial design. For the monetary reward task, we used a control group, whereas the food viewing task followed a within-subject design.

### Participants

Patients were recruited from major clinics specialized in adult eating disorders in the Netherlands. We applied the following inclusion criteria: a clear primary diagnosis of AN (restricting or purging subtype) based on the DSM-IV, confirmed by a psychiatric interview conducted by an independent physician; illness duration of ≥ 10 years; and BMI < 15. Additionally, patients must have shown no response to ≥ 2 typical modes of treatment, including one hospital admission or inpatient treatment in an eating disorder specialized clinic. They should also have exhibited substantial functional impairment according to the DSM-IV criterion C and a Global Assessment of Function-score (GAF-score) of ≤ 45 for ≥ 2 years. The exclusion criteria are described previously [1]. This resulted in four female AN-patients who were enrolled from 2016 to 2020. Additionally, data from six healthy control subjects were included from previous (neuroimaging) projects of our department. The control subjects and their first-degree relatives had to have negative lifetime histories of psychiatric illness, as evaluated by SCID I and SCID II interviews. The controls were matched for sex (all female), but not for age (M = 54; SD = 4.7). The control subjects had no DBS-electrodes implanted and but were scanned at three different time-points.

### Procedure

We conducted bilateral stereotactic implantation of DBS electrodes in the ventral anterior limb of the capsula interna (vALIC). Following our earlier DBS studies, we distinguished four sequential study phases: preoperative (T-1), surgery (T0), optimization (3–9 months; T1-T2) and maintenance (12 months; T2-T4). After screening at T-1, bilateral DBS electrodes were implanted in the vALIC at T0. We turned on and optimized DBS settings from T1 to T2, and followed patients up to T4. During the study, patients received standard medical and psychiatric care, which included regular visits with a nurse-practitioner and a psychiatrist. No major psychopharmacological adjustments were made.

### Measurements

**Fig. 1:**
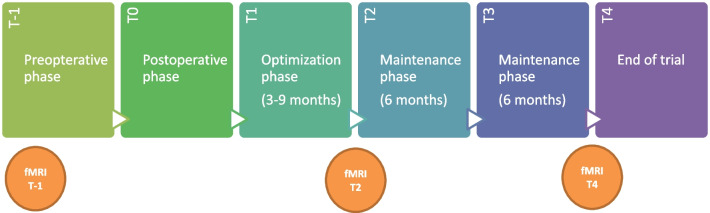
The temporal phases of the DBS treatment with the fMRI time-points at T-1, T2 and T4

We conducted fMRI scans at three time-points: 1) pre-operatively at T-1 as a baseline measurement, 2) at the end of the optimization period (T2, to investigate short-term effects of stimulation), and 3) at 12 months after ending the optimization period (T4, at the end of the maintenance phase) (See Fig. 1). For controls, fMRI was conducted at three time-points as well, matching the time intervals of the AN group, however they did not receive DBS treatment. In addition to neurophysiological measures, we closely monitored patients clinically and psychologically during follow-up by means of BMI and psychiatric symptom questionnaires [1].

### Tasks

The monetary reward task used motivational rewarding stimuli [31] in the form of cues predicting a rewarding, neutral or loss outcome. Each condition consisted of three different levels regarding the magnitude of the outcome to motivate participants and enhance reward uncertainty. The presentation of the cues, which constituted the reward anticipation phase, was followed by a target to which participants had to respond as fast as possible. After responding, participants received feedback on their final monetary rewarding, neutral or loss outcome. In case of a positive monetary rewarding outcome, the actual amount of money was provided. Time to respond was limited by individual reaction times collected before the experiment to create equal performance across participants. Trial conditions were counterbalanced at random order (36 trials per condition) and trial durations were randomly varied (6-10 s per trial). The total duration of the task was 14 min.

During the food viewing task, subjects were presented with images of non-food, high-calorie food and low-calorie food using a paradigm similar to previous studies conducted on both healthy subjects and anorexia patients [21, 33]. We used standardized food and neutral pictures from the database developed by Charbonnier e.a. [34]. The images were presented over six blocks with a duration of 30 s per block. The blocks were pseudorandomized and alternately consisted of 10 images from one condition (non-food, high-calorie or low-calorie). Between blocks, subjects received questions about their desire to eat and level of anxiousness. The total duration of the task was 12 min.

### Data acquisition

The data were collected using the 1.5 Tesla Siemens Avanto scanner at the Amsterdam UMC (location AMC, department of radiology). To minimize exposure of DBS electrodes to the pulsed radiofrequency field, a transmit and receive head coil was used. DBS was turned off before patients entered the scanner, and the specific absorption rate was limited to 0.1 W/kg. During task performance, the blood oxygen level dependent (BOLD) MRI signal was acquired using a functional T2*-weighted two-dimensional echo-planar imaging sequence (TR = 2000 ms, echo time = 30 ms, flip angle = 90°, field of view = 230 × 230 mm, matrix = 64 × 64, 25 slices, slice thickness = 4 mm, slice gap = 0.4 mm).

### Data analysis

The MRI data were analyzed using SPM12 (version 6685, Wellcome Trust Centre for Neuroimaging, London, UK) and MATLAB (version R2014a, The MathWorks Inc., Natick, MA, USA). fMRI data preprocessing consisted of realignment, slice-time correction, normalization to Montreal Neurological Institute (MNI) space, resampling to 2 × 2 × 2 mm^3^ and spatial smoothing with a Gaussian-kernel of 8 mm full width at half maximum (FWHM). A high pass filter of 1/128 Hz was applied to the data and serial correlations were accounted for using the autoregressive AR(1) modelling. A general linear model (GLM) was used to model the conditions of interest (see below), convolved with a hemodynamic response function (HRF) using 3 regressors related for either the reward task (neutral, reward, loss) or the food task (non-food, high-calorie and low-calorie food), and 6 additional regressors to account for head motion parameters. 

For the reward task, the conditions of interest (reward > neutral; loss > neutral) were specified in first-level modelling, including the onset and duration of anticipation cues. A second-level full factorial design was created, combining time (T-1, T2, T4), group (AN, controls) and condition (reward > neutral, loss > neutral). The interaction effects between group and time were investigated for both reward and loss anticipation separately.

For the food task, the non-food, high-calorie food and low-calorie food conditions were modeled as box-car regressors at first-level, and a second-level repeated measures ANOVA design was created,combining time (T-1, T2, T4), and condition (high-calorie food > non-food, low-calorie food > non-food). The interaction effects for time and condition were investigated within subjects. The results for both tasks were masked to exclude voxels with DBS-related signal dropout in the normalized EPI scans.

First, an assessment was made of significant differences between the two post-operative sessions. As we did not observe significant group by time interactions when comparing the two post-DBS timepoints (T2, T4), we created a contrast in which both post-DBS time points were equally weighed against the pre-DBS time point (T-1) to improve statistical power.

A region of interest (ROI) analysis was performed for the ventral striatum (VS) and the medial orbitofrontal cortex (mOFC). These regions were chosen *a-priori* based on their strong relation to reward processing [13], their role in AN pathology and their previous use in AN-fMRI-studies [14–16]. Furthermore, the VS was also the target of DBS. The VS was based on peak-coordinates from a previous study using a similar monetary reward task [17]. Supplementary figure S1 illustrates that the ROI in the VS is located outside the regions that are affected by signal dropout from the DBS electrode (Additional file 1: figure S1). The mOFC was based on the IBASPM 71 atlas in the WFU Pickatlas toolbox in SPM12. Voxel-wise statistical tests were family-wise error (FWE) rate corrected for multiple comparisons (*p* < 0.05) across the whole-brain at the cluster level using a height-threshold of *p* < 0.001 or for ROIs at the voxel level using a small volume correction (SVC) [35].

## Results

### Sample characteristics

Four patients with treatment-refractory AN were included in this study and underwent DBS of the vALIC, during 12 months follow-up. Mean age was 39 (SD = 10) and illness duration 21 years (SD = 3). All patients were female and suffered from binge-purging subtype of AN. Average BMI at baseline was 12.5 (SD = 1.0) kg/m^2^, indicating extremely severe AN. All patients suffered from psychiatric comorbidities. Two patients were diagnosed with personality disorder not otherwise specified (PD-NOS), one patient was diagnosed with major depressive disorder (MDD) and one patient was diagnosed with both PD-NOS and MDD. All demographic characteristics are shown in Table 1.

**Table 1:** Patient’s demographics

	Sex	Age at onset (years)	Age at surgery (years)	Illness duration (years)	Anorexia subtype	BMI (screening)	BMI (historic low)	Psychiatric comorbidities	Psychiatric medication at surgery
Patient 1	f	15	32	18	Purging	12.4	9.5	GAD, Depression, Personality Disorder NOS	Aripiprazole, Quetiapine, Oxazepam
Patient 2	f	15	39	24	Purging	11.2	10.2	SUD*, Personality Disorder NOS	Aripiprazole, Quetiapine, Oxazepam
Patient 3	f	14	33	19	Purging**	13.4	10.6	Depression, OCD, BPD	Venlafaxine, Clorazepate, Oxazepam, Temazepam
Patient 4	f	27	53	24	Purging	13.1	9.7	Personality Disorder NOS	Citalopram, Alprazolam, Zolpidem
Mean (SD)		18 (6)	39 (10)	21 (3)		12.5 (1.0)	10.0 (0.5)		

BMI = Body Mass Index, f = female, GAD = Generalized Anxiety Disorder, OCD = Obsessive Compulsive Disorder, NOS = Not Otherwise Specified, SUD = Substance use disorder and BPD = Borderline Personality disorder

^*^The substance in question was alcohol, at screening this was in remission

^**^Important to note that for purging this patient used ± 50 Bisacodyl

At time-point T1 for the AN-patients, monopolar DBS at the middle two contacts was switched on, with a pulse width of 90μsa and afrequency of 130 ms, at a mean voltage of 3.0 V (2.5–3.5 V). The mean voltages at T2, T3 and T4 were 3.8 V (3.0–5.0 V), 3.8 V (3.0–4.5 V) and 3.8 V (2.7–4.8 V) respectively. Adjustment of the stimulation intensity occurred in steps of 0.5 V, with later fine-tuning in steps of 0.1 V. Pulse width and frequency remained unchanged during the study.

**Fig. 2:**
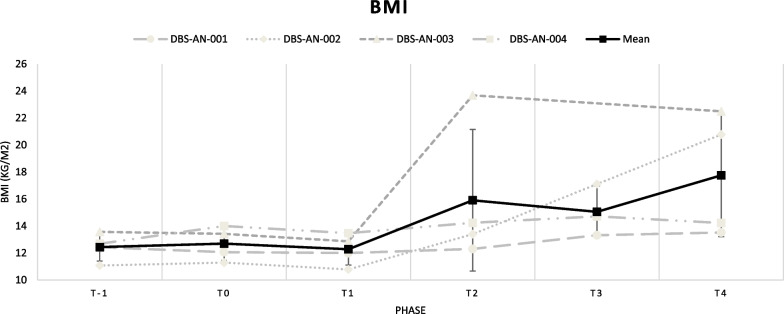
Time course of DBS-induced BMI (fixed effects ± SE). Linear mixed model analyses showed a significant linear effect of time on BMI (43.16 ± 15.96, CI 95% 9.07–77.25, t = 2.704, *p* = .017). This figure was originally published in Oudijn et al. [1]

We previously published the primary outcomes of this study [1]. In our findings, we observed a significant increase in BMI at the end of the follow-up period (5.32 kg/m^2^; + 42.8%; *p* = 0.017). This increase in BMI was primarily seen in two out of the four patients (subject 2 and subject 3) (see Fig. 2). Additionally, we found significant decreases in psychiatric symptom questionnaire scores, which measured eating disorder symptoms, depression and anxiety.

During the intraoperative period, no adverse events were observed. However, we recorded 28 severe adverse events (SAEs), with two being probably related((hypo)manic symptoms) and nine being possibly related (self-destructive behavior)to the intervention. It is worth noting that most of the SAEs were related to the (somatic) severity of AN rather than DBS (n = 11).

### Neuroimaging results

**Fig. 3:**
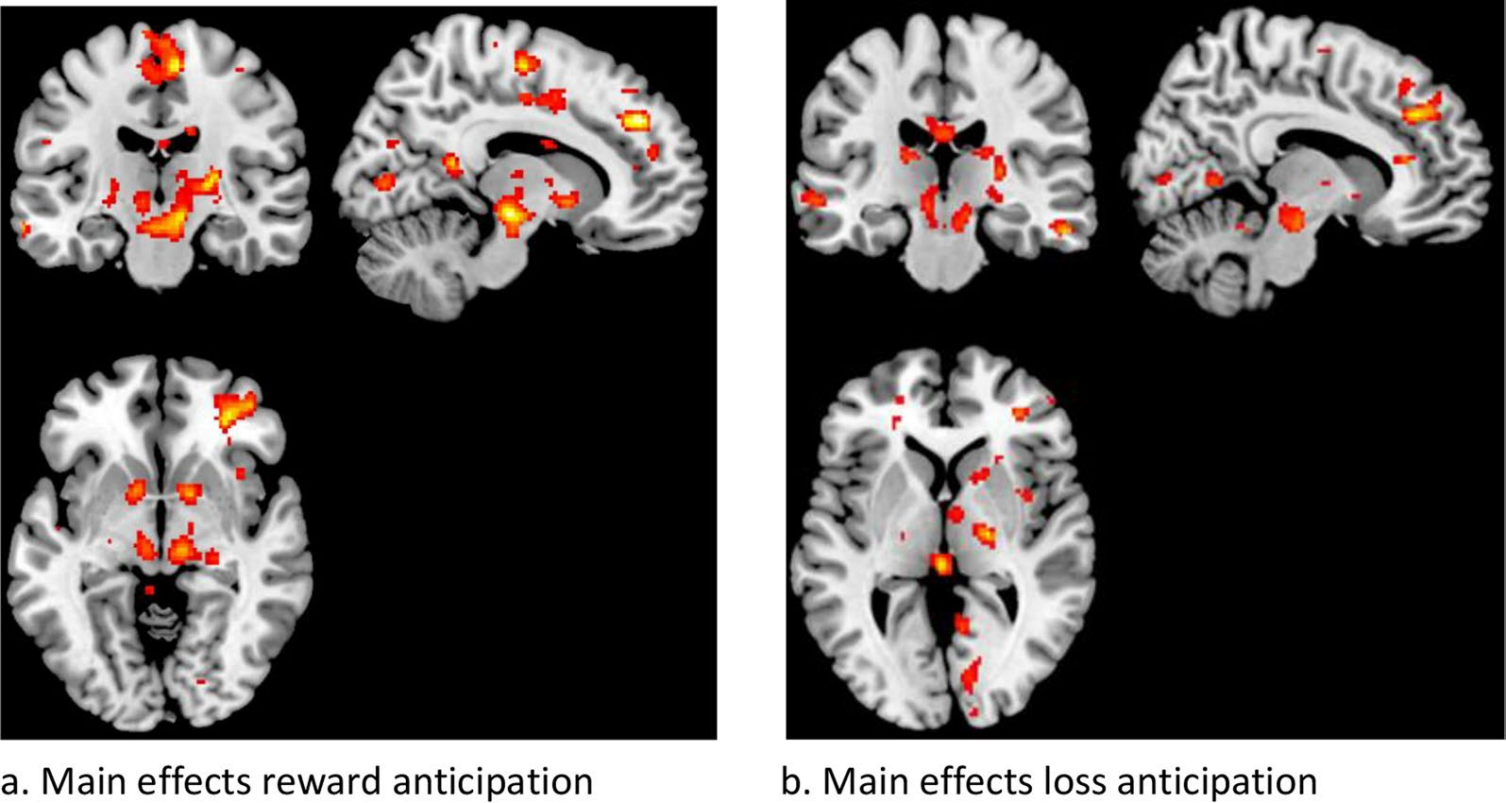
Main effects of task illustrated for AN and HC combined at all timepoints, using a threshold of *p* < 0.01 uncorrected for visualization purpose

*Monetary incentive delay task*—The MID task revealed a main effect of reward anticipation, showing higher activation n the bilateral thalamus, ventral striatum, insula, medial prefrontal cortex and brain stem during the anticipation of reward compared to neutral cues (See Fig. 3a). The threshold of *p* < 0.01 uncorrected for visualization purposeswas used. Similarly, the main effect of loss anticipation also showed activation in the same regions (See Fig. 3b).

**Fig. 4:**
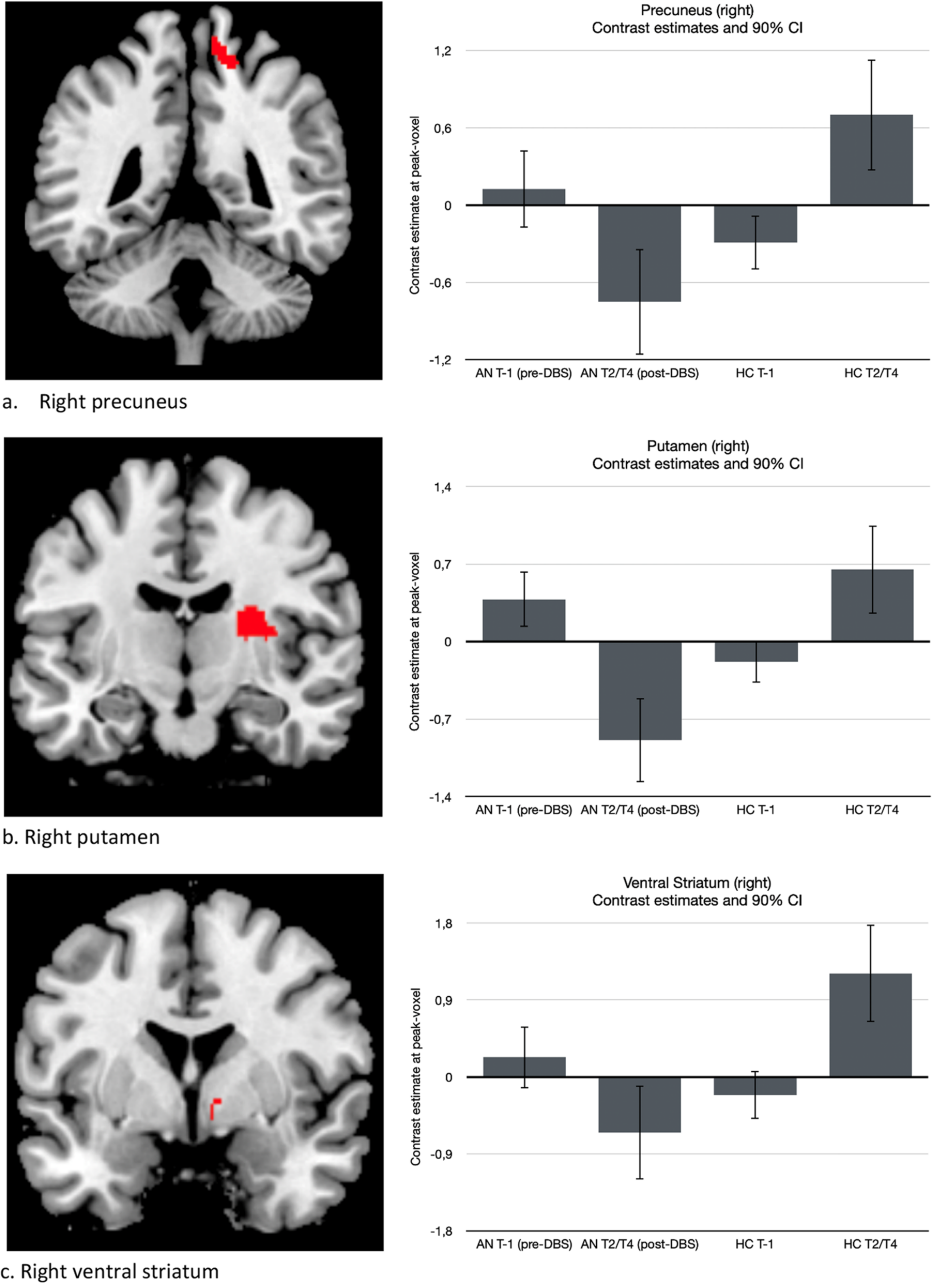

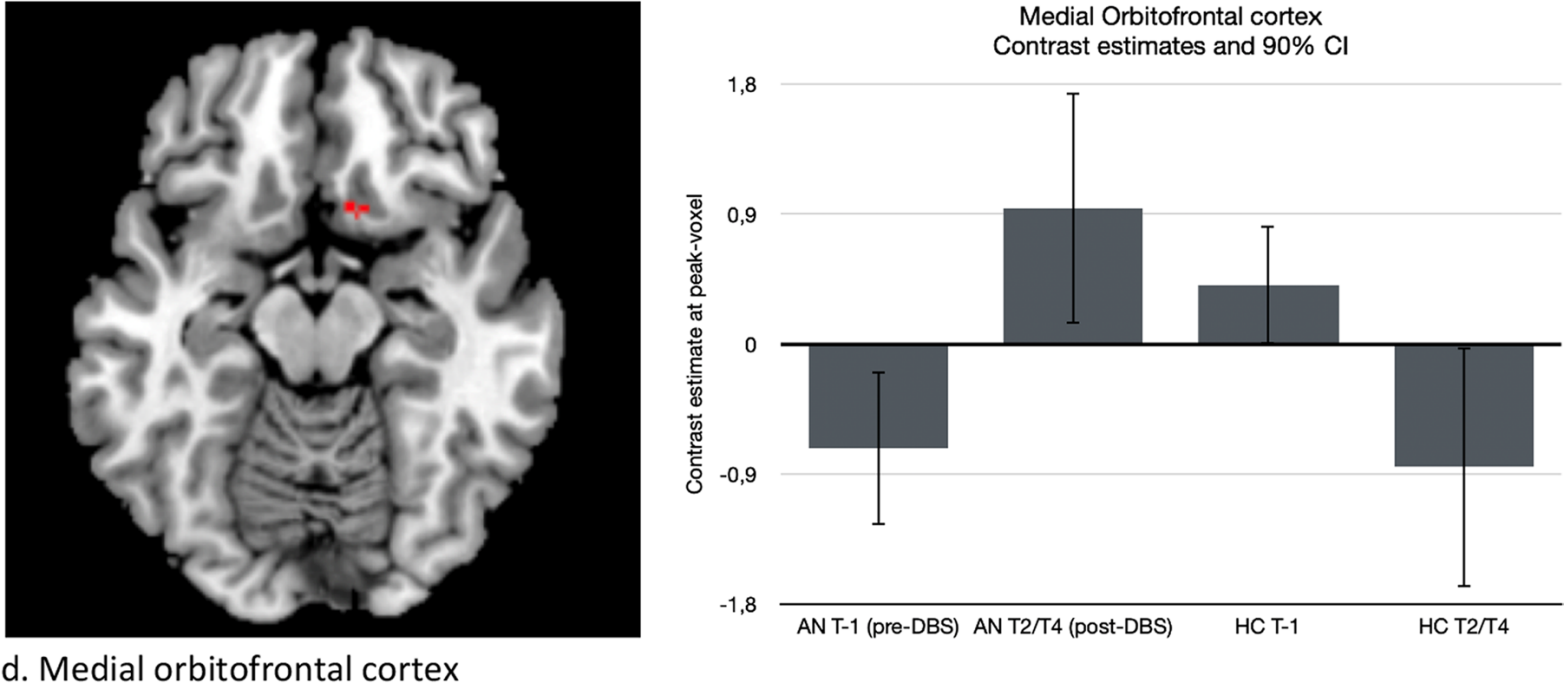
Significant interaction effects for reward anticipation for each anatomical region displayed at *p* < 0.001, uncorrected (left) and corresponding bar plots displaying the contrast estimates for the interactions at the peak voxels (right)

At baseline (pre-DBS, T-1), no significant differences in *reward anticipation* were found between AN and HC. However, significant group (AN and HC) by time (pre- and post-DBS) interactions were found in the right precuneus (xyz = 16,-44,60; Z = 3.99; *p*-FWE = 0.035; cluster size = 132), right dorsal putamen (xyz = 26,-10,16; Z = 4.69; *p*-FWE = 0.025; cluster size = 169), right ventral striatum (xyz = 8,0,-6; Z = 3.64; *p*-SVC = 0.030), and mOFC (xyz = -2,32,-22; Z = 3.55; *p*-SVC = 0.043) (See Fig. 4 and Table 2). Follow-up testing revealed that the interaction effects in the precuneus, putamen and VS could be explained by lower activation for AN post-DBS compared to pre-DBS, whereas in HC, there was higher activation (right precuneus (xyz = 12,-42,64; Z = 3.97; *p*-FWE = 0.058; cluster size = 135), right putamen (xyz = 26,-10,16; Z = 4.08; *p*-FWE = 0.020; cluster size = 85) and right VS (xyz = 8,0,-6; Z = 3.50; *p*-SVC = 0.041). The interaction effect in the mOFC could be explained by higher activation for AN post-DBS compared to pre-DBS, while in HC, activation was lower (xyz = -2,32,-22; Z = 3.55; *p*-SVC = 0.043).

**Table 2:** Significant interaction effects for reward anticipation, pre- and post-DBS are compared for AN versus HC

Anatomical region	Time point	Effect ↑ increase ↓ decrease	Cluster size	MNI-coordinates			Statistics	
				x	y	z	Z-score	*p*-value
Precuneus R	Post-DBS	AN ↓	132	16	− 44	60	3.99	0.035
Putamen R	Post-DBS	AN ↓	169	26	− 10	16	4.69	0.025
VS R	Post-DBS	AN ↓	N/A	8	0	− 6	3.64	0.030*
mOFC	Post-DBS	AN ↑	N/A	− 2	32	− 22	3.55	0.043*

The direction of the effect is illustrated by an increase or decrease for the AN group post-DBS

*p-value after small volume correction (SVC)

**Fig. 5:**
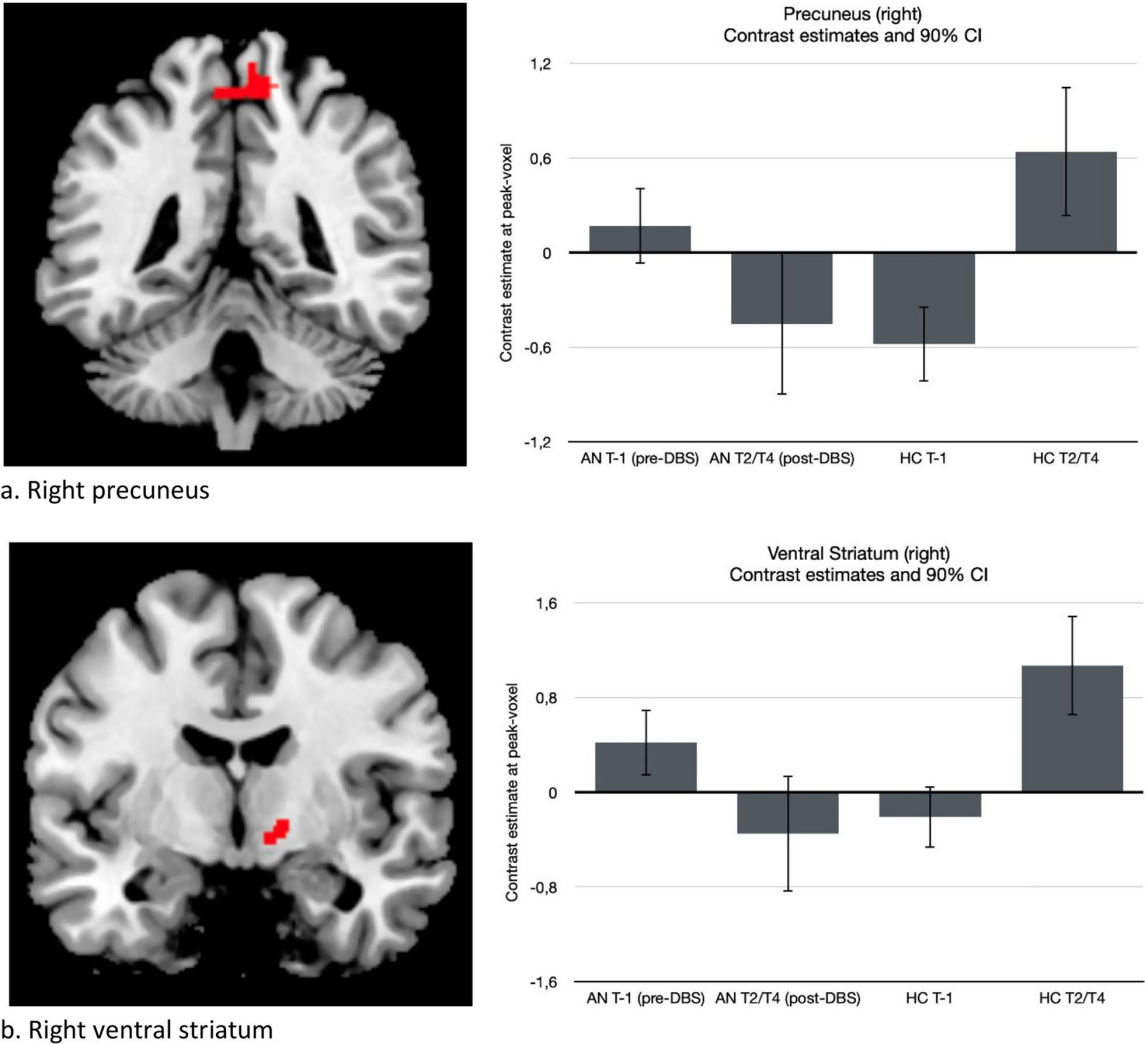
Significant interaction effects for loss anticipation for each anatomical region displayed at *p* < 0.001, uncorrected (left) and corresponding bar plots displaying the contrast estimates for the interactions at the peak voxels (right)

At baseline (pre-DBS), no significant differences for *loss anticipation* were found between AN and HC. However, significant group (AN and HC) by time (pre- and post-DBS) interactions were observed in the right precuneus (xyz = 10,-44,64; Z = 4.15; *p*-FWE = 0.007; cluster size = 222) and the right VS (xyz = 12,-4,-4; Z = 3.75; *p*-SVC = 0.021) (See Fig. 5 and Table 3). These interactions could both be explained by significantly lower activation for AN post-DBS compared to pre-DBS, while activations were higher in the HCs, especially in the right precuneus (xyz = 10,-44,64; Z = 4.15; *p*-FWE = 0.007; cluster size = 222) and right VS (xyz = 12,-4,-4; Z = 3.89; *p*-SVC = 0.012).

**Table 3:** Significant interaction effects for loss anticipation, pre- and post-DBS are compared for AN versus HC

Anatomical region	Time point	Effect ↑ increase ↓ decrease	Cluster size	MNI-coordinates			Statistics	
				x	y	z	Z-score	*p*-value
Precuneus R	Post-DBS	AN ↓	222	10	− 44	64	4.15	0.007
VS R	Post-DBS	AN ↓	91	12	− 4	− 4	3.89	0.012*

The direction of the effect is illustrated by an increase or decrease for the AN group post-DBS

*p-value after small volume correction (SVC)

*Food viewing task*—No significant interactions were found between the conditions of high-calorie and low-calorie food images. Therefore, both conditions were combined as one food condition for further comparisons. Additionally, no significant interactions were found between the different time points, indicating that we did not find evidence for altered responses for food viewing between the pre- and post-DBS time points.

## Discussion

The present study investigated the neurobiological effects of vALIC DBS in AN patients using two tasks: the monetary incentive delay task and a food viewing task. The monetary incentive delay task was usedto study non-illness-specific food related reward-processing, while the food viewing task focused on more illness-specific reward processing. We hypothesized that AN-patients would show higher activation than HCs in reward-related brain areas during the monetary tasks pre-DBS, especially with losses-indicative of the heightened sensitivity to punishment in AN- and that this activation would normalize following DBS. We expected to find similar effects in the illness-specific task.

In contrast to other studies [21, 22], we did not observe differences between AN patients and controls at baseline. However, we did find changes in the frontostriatal circuit during reward and loss anticipation in AN patients, with a decrease in right precuneus, right putamen and right VS activation, and an increase in mOFC activation following DBS. Conversely, increases in activation were seen in the HC group over time. These findings indicate a difference in response in het AN group after treatment with DBS.

The VS mediates reward, reinforcement and motivational salience. In response to both monetary and visual food cues, AN patients show hypoactivity of the striatum [36]. Our hypothesis was that in AN, there would be hyperactivity of the reward system in response to illness-compatible cues (including punishment) and less increased activity of the reward system in response to immediate rewards. The decreased activation of the right VS following DBS suggests a normalization of aberrant hyperactivity of the VS to reward and punishment, possibly indicating restoration of goal-directed rather than illness-compatible behavior.

The mOFC, a subregion of the ventromedial prefrontal cortex, is linked to cognitive flexibility and context-specific responding, encoding emotional and reward value in decision-making. The fact that we found an increase in mOFC activation following DBS seems contradictory to the hypothesis that excessive cognitive control would decrease after treatment. One possibility is that the mOFC activity increases after DBS due to changes in other parts of the reward circuitry, leading to increased contingencies. Another explanation for the increase in mOFC activation following DBS could be that patients have improved in valuating outcome relative to context.

The above findings are in line with a study on DBS and OCD that showed that DBS targeted at the nucleus accumbens (NAc; part of the VS) normalized NAc activity and restored pathological network activity [27].

The precuneus is implicated in self-processing and agency. Decreased activity of the precuneus after DBS might indicate an increase in non-self-referential goal-directed behavior and a restoration of the brain default network.

Despite our hypothesis of increased activation in reward-related areas in the food viewing tasks with explicit rating, especially with high-calorie food pictures compared to low-calorie pictures, the results did not show any significant pre- and post DBS differences in activation in response to all pictures. This could be possibly due to the small sample size and the lack of a control group.

Both the literature and our study support the hypothesis regarding the involvement of the reward circuitry in the pathogenesis of anorexia nervosa. However, reward processing is heterogeneous and is influenced by emotions such as fear and body image perception. Furthermore, it remains unclear whether aberrant activation in specific areas is related to reward or punishment, which should be examined in future research. Nevertheless, the correspondence of our results for reward and punishment anticipation suggests that the effects of DBS may be generic for motivational behavior.

In healthy controls, hunger enhances sensitivity to and motivation for reward. However, remitted AN patients do not show the same increased activation in reward salience circuitry during the processing of immediate reward when hungry, nor do they show increased activation in the cognitive control circuitry when satiated, as observed in healthy controls [22]. We conducted the fMRI scans in a non-fasted state. However, it is difficult to assess whether a non-fasted state in AN patients equates to being sated, and vice versa. We did assess hunger with a visual analogue scale in the AN group, but all patients rated their hunger as low to absent, regardless of previous food intake or fasting. As a result, we could not determine whether hunger, as an enhancer of motivational drive, influenced the outcomes this study.

The differences in outcomes in our study compared to other imaging studies [4, 29], which found reduced activity of the subcallosal and anterior cingulate, and hyperactivity of parietal structures, as well as decreased activity in the frontal lobe, hippocampus, lentiform nucleus, left insula, and left subcallosal gyrus after DBS, could be explained by the fact that those studies used resting-state PET instead of task-based fMRI. The differences in results could, therefore, be attributed to investigating tonic activity (PET) versus phasic activity (fMRI), which may occur at the same time.

Limitations of our study include the small sample size, which is a result of the highly specific setting and population of the study (an experimental intervention study in physically compromised patients), leading to very low power. Moreover, the food viewing task was even more underpowered due to the lack of a control group, which precluded testing for group x time interactions. Due to the small sample we were unable to link changes in activation patterns to clinical effect, symptoms and/or behavior.

### Future directions

To gain more insight into the involvement of the reward circuitry in the etiopathogenesis and treatment of AN, larger neuroimaging studies should be conducted. It is also of great importance to develop ways to link neuroimaging data to clinical/behavioral data. Future studies on DBS in AN should include (functional) neuroimaging using more disease-specific tasks to better understand the differences in reward response to clinical features of AN. This could contribute to the knowledge of the etiopathological mechanisms in AN and the functional effects of DBS. Forming an international collaboration to conduct fMRI on a larger group of AN patients treated with DBS or a transdiagnostic approach comparing DBS in AN with DBS in OCD or MDD would be valuable for investigating possible individualized DBS-targeting.

## Conclusion

The aim of our fMRI study was to investigate the effects of reward-circuitry targeted DBS in AN patients. We conducted functional MRI scans pre and post DBS and found differences in the response of reward-related brain areas post DBS. This supports the hypothesis that the reward circuitry is involved in the pathogenesis of anorexia nervosa and that DBS influences aberrant network activity. Further neuroimaging studies on DBS in AN with larger sample sizes, a more disease-specific paradigm, and a sham control condition should be considered.

### Supplementary Information

**Additional file 1: Figure S1**: Illustrates that the ROI in the ventral striatum is located outside the regions that are affected by signal dropout from the DBS electrode.

## Data Availability

The datasets used and/or analysed during the current study are available from the corresponding author on reasonable request.
