# Supplementary material for: Neural effects of deep brain stimulation on reward and loss anticipation and food viewing in anorexia nervosa: a pilot study
Source: J Eat Disord. 2023 Aug 21;11:140. doi: 10.1186/s40337-023-00863-3 (PMC10440869; doi:10.1186/s40337-023-00863-3)
Supplement: Supplementary file 1 — Additional file 1: Figure S1: Illustrates that the ROI in the ventral striatum is located outside the regions that are affected by signal dropout from the DBS electrode. [file 40337_2023_863_MOESM1_ESM.docx]

**Supplement 1**


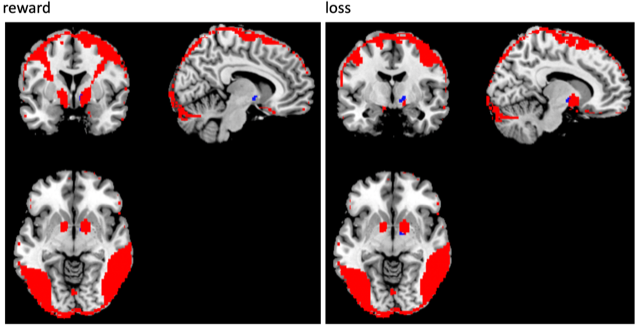


**Figure S1.** This figure illustrates that the ROI in the ventral striatum (blue) is located outside the regions that are affected by signal dropout form the DBS electrodes (red).
